# Anti-oxidant, anti-apoptotic, and protective effects of myricitrin and its solid lipid nanoparticle on streptozotocin-nicotinamide induced diabetic nephropathy in type 2 diabetic male mice

**DOI:** 10.22038/IJBMS.2023.22499

**Published:** 2023

**Authors:** Akram Ahangarpour, Ali Akbar Oroojan, Layasadat Khorsandi, Maryam Kouchak, Mohammad Badavi

**Affiliations:** 1 Department of Physiology, Faculty of Medicine, Diabetes Research Center, Health Research Institute, Ahvaz Jundishapur University of Medical Sciences, Ahvaz, Iran; 2 Department of Physiology, Faculty of Medicine, Cellular and Molecular Research Center, Student Research Committee, Ahvaz Jundishapur University of Medical Sciences, Ahvaz, Iran; 3 Department of Physiology, Faculty of Medicine, Dezful University of Medical Sciences, Dezful, Iran; 4 Department of Anatomical Sciences, Faculty of Medicine, Cellular and Molecular Research Center, Ahvaz Jundishapur University of Medical Sciences, Ahvaz, Iran; 5 Department of Pharmaceutics, Faculty of Pharmacy, Nanotechnology Research Center, Ahvaz Jundishapur University of Medical Sciences, Ahvaz, Iran; 6 Department of Physiology, Faculty of Medicine, Physiology Research Center, Ahvaz Jundishapur University of Medical Sciences, Ahvaz, Iran

***To the editor***

Diabetic nephropathy (DN) is one of the diabetes-associated complications that develop in both type 1 and type 2 diabetes. The underlying pathophysiological mechanisms leading to DN are similar in type 1 and type 2 diabetes (1). The most common features of DN include a variable period of hyperfiltration, which is followed by a persistent, progressive decline in glomerular filtration rate (GFR) that starts to happen once there is noticeable proteinuria (2). Generally, GFR increases transiently in the early stages of diabetes, but due to renal damage, GFR eventually decreases (2-5). The pathophysiology of DN is complex and many factors affect its progression and severity. Whether GFR increases or decreases in diabetic subjects depends on many factors, such as the animal species, strain, model used for DN induction, duration of the study, severity of diabetes and hyperglycemia, etc., and the results reported by different investigators are not similar. Indeed, both increased and decreased GFR are indicators of DN, increased GFR due to hyperfiltration is a marker of early stages of DN, and decreased GFR is a marker of late stages of DN (5, 6). For example, Zheng *et al*. showed that in OVE26 mice, a transgenic model of type 1 diabetes, GFR increased significantly from 2 to 3 months of age and then decreased significantly from 5 to 9 months (3). Similar to our study, Saravanan and Pari (2016) reported that only 5 weeks after diabetes induction in mice using a high-fat diet creatinine clearance (GFR) significantly decreased compared with normal control mice (7). Also, albuminuria is the other marker of DN that is a predictor of decline in GFR (5). Consistent with these findings, in the present study we observed that albumin excretion increased in diabetic mice compared with normal control mice. So, it can be concluded that in our study, the induction of the DN model was successful. 

 In diabetic patients, loss of glucose through the kidneys leads to hyperosmotic urination, which eventually results in loss of water and electrolytes (8). Insulin stimulates Na+ transport in all parts of the nephron, including the proximal tubule, the thick ascending limb, and the distal tubule and collecting ducts, therefore exerting an anti-natriuretic effect (9). Insulin also is needed for the proper functioning of Na+/K+ pumps. Wilson *et al*. reported that during diabetes the expression of genes involved in Na+ and K+ reabsorption decreases in the different parts of the nephron (10). So, it can be concluded that elevated urinary Na^+^ in diabetic mice can be the result of the diminished insulin actions in these animals. Rosas-Martínez *et al*. reported that in both type 1 and type 2 diabetic rats fractional excretion of Na+ (FE Na+) increased in diabetic animals compared with normal control animals (11). Consistently, in this study, we observed a significant increase in FE Na+ in diabetic mice. 

Indeed, increased glomerular hydrostatic pressure activates the renin-angiotensin-aldosterone system, and not vice versa (12). 

## Conflicts of Interest

None. 
